# Evaluation of CXCL12 Diagnostic Performance in Tick-Borne Encephalitis and Other Central Nervous System Infections

**DOI:** 10.3390/ijms27125549

**Published:** 2026-06-19

**Authors:** Paulina Świętoń, Jakub Słoń, Anna Moniuszko-Malinowska, Ewelina Kruszewska, Sambor Grygorczuk

**Affiliations:** 1 Student Scientific Society at the Department of Infectious Diseases and Neuroinfections, Medical University of Bialystok, ul. Żurawia 14, 15-540 Białystok, Poland; kubaslon23@gmail.com; 2 Department of Infectious Diseases and Neuroinfections, Medical University of Bialystok, ul. Żurawia 14, 15-540 Białystok, Poland; anna.moniuszko-malinowska@umb.edu.pl (A.M.-M.); ewelina.kruszewska@umb.edu.pl (E.K.); sambor.grygorczuk@umb.edu.pl (S.G.)

**Keywords:** chemokine CXCL12, neuroinfection, tick-borne encephalitis, pleocytosis

## Abstract

CXCL12 is a chemokine acting via CXCR4 that plays a key role in inflammatory processes by regulating leukocyte migration and immune responses, making it an important focus of research in neuroinfections. In this study, the CXCL12 chemokine was evaluated as a potential diagnostic marker of different neuroinfections with particular emphasis on tick-borne encephalitis (TBE). A group of 214 patients with confirmed meningitis and/or encephalitis was included and divided according to etiology into TBE, aseptic meningitis, neuroborreliosis, and purulent meningitis. CXCL12 concentration and other inflammatory parameters were measured in cerebrospinal fluid (CSF). CXCL12 levels were compared with those of controls (*N* = 25) and analyzed statistically. In addition, serum was used to determine albumin concentrations. CXCL12 concentrations were significantly higher in patients with neuroinfections compared to controls and showed good diagnostic performance in the overall study group (AUC = 0.791), with a more moderate diagnostic performance observed in individual etiological groups, except in the purulent meningitis group, where the effect was not statistically significant, likely due to the small sample size. CXCL12 also demonstrated some utility in differentiating between specific etiologies; however, this effect was limited. Better diagnostic performance was observed when CXCL12 was combined with pleocytosis in a composite model differentiating between the TBE group and aseptic meningitis (AUC = 0.761). The presented results indicate the role of CXCL12 in neuroinflammation while simultaneously highlighting its potential in the development of novel diagnostic approaches for viral neuroinfections. Despite higher levels in TBE, its standalone diagnostic value is limited; however, it may enhance diagnostic accuracy when combined with other markers such as pleocytosis.

## 1. Introduction

Although multiple studies have investigated the immune response in central nervous system (CNS) infections, the role of individual chemokines in the pathogenesis of neuroinfections is not yet fully understood. The chemokine CXCL12 (stromal cell-derived factor-1) is a molecule with a broad spectrum of chemotactic activity toward mononuclear cells, acting through the specific receptor CXCR4 [1,2]. The CXCL12–CXCR4 signaling axis plays a role, among others, in inflammatory processes, regulating the blood–brain barrier (BBB) integrity, and in regulating leukocyte migration into the CNS, making it a significant subject of research in neuroinfections [3,4,5,6,7].

Although the biological functions of CXCL12 have traditionally been attributed primarily to signaling through CXCR4, increasing evidence indicates that ACKR3, an atypical receptor for CXCL12, also plays an important regulatory role within the CXCL12 signaling axis. Moreover, the CXCL12/CXCR4/ACKR3 axis represents a highly promising therapeutic target, as its modulation may influence processes such as neuroinflammation, remyelination, and neurodegeneration [8,9,10]. The biological effects of chemokine signaling appear to depend on multiple factors, including the type of CNS disorder, receptor expression patterns, local chemokine concentration, cellular and anatomical localization within the CNS, integrity of the BBB, as well as the duration of the inflammatory process [8]. Notably, CXCL12 has been shown to act bidirectionally by having both pro-inflammatory and protective effects depending on the pathological context and its local concentration [5]. These observations highlight the complexity of CXCL12-mediated signaling and underscore the need for further studies evaluating its role in CNS infections.

Our studies particularly focus on *Flavivirus* tick-borne encephalitis (TBE), pathogenesis of which is largely driven by immune-mediated mechanisms, with inflammatory processes playing a crucial role in disease progression and neurological damage [11,12,13]. The disease is characterized by a wide spectrum of clinical manifestations, ranging from mild meningitis to severe meningoencephalomyelitis, reflecting substantial variability in host immune response [11,14,15]. It is accompanied by an influx of a dynamic population of fluctuating leukocytes that can be observed in the cerebrospinal fluid, dominated by CD4+ T lymphocytes, with a notable contribution from CD8+ T lymphocytes and neutrophils [16,17]. Given that, chemokines such as CXCL12 may be particularly relevant in the pathophysiology of TBE. 

Knowledge about differences in chemokine concentrations and their diagnostic performance across neuroinfections of different etiologies is still insufficient. Importantly, it remains unclear whether CXCL12 levels may provide clinically useful information regarding disease severity, its progression in time, or assist in the differential diagnosis of neuroinfections of diverse etiologies. Furthermore, the relationship between CXCL12 and established markers of neuroinflammation, such as pleocytosis, lymphocyte count, and BBB dysfunction, requires further investigation. 

However, several observations indirectly suggest that CXCL12 may hold clinical value in this context. Elevated CXCL12 concentrations have been demonstrated in the CSF of patients with both TBE and neuroborreliosis [18,19]. These studies did not evaluate whether CXCL12 levels correlate with disease severity or clinical outcome, leaving its diagnostic and prognostic potential unexplored. However, experimental models of neuroinfections provide direct evidence linking CXCL12 to disease severity. In a mouse model of West Nile virus encephalitis, modulation of the CXCL12–CXCR4 axis directly affected leukocyte trafficking, viral clearance, and host survival [20,21]. Beyond CNS infections, CXCL12 has also demonstrated potential clinical utility as a biomarker. In COVID-19, serum CXCL12 concentrations correlated with clinical severity [22]. Elevated CXCL12 serum levels have been associated with disease severity and mortality in patients with severe sepsis and septic shock, suggesting prognostic value of this chemokine [23]. Taken together, these findings from both CNS and systemic infections indicate that CXCL12 may serve as a clinically informative biomarker and warrant its further dedicated investigation in neuroinfections such as TBE.

The aim of this study was to evaluate the role and diagnostic utility of the CXCL12 chemokine in patients with central nervous system infections of different etiologies, with a particular focus on TBE.

## 2. Results

### 2.1. Study Group

The whole study group consisted of 214 patients (median age 45; 77 females and 137 males). The study group was divided according to etiology into TBE (*N* = 159) (median age 46; 53 females and 106 males), aseptic meningitis, which included also herpetic encephalitis (*N* = 41) (median age 35; 18 females and 23 males), neuroborreliosis (NB) (*N* = 9) (median age 60; 4 females and 5 males), and purulent meningitis including streptococcal and pneumococcal (*N* = 5) (median age 56; 2 females and 3 males). The control group consisted of 25 patients (median age 39; 18 females and 7 males). No statistically significant difference in CXCL12 levels was observed between sexes and there was no statistically significant correlation between CXCL12 concentrations and age (*p* > 0.05 in both). Because the median age differed between the control group and the study groups, we assessed the age distribution across groups and confirmed a statistically significant difference. Therefore, a multivariable linear regression analysis including both age and case–control status was conducted. Age was not a significant predictor of CXCL12 levels in any of the models, whereas the group effect remained significant, indicating that the observed differences were independent of age.

### 2.2. Diagnostic Value of CSF CXCL12 Concentration and Pleocytosis

Median CXCL12 concentration (pg/mL) for the whole study group was 2201.06, while the median pleocytosis (cells/µL) was 102.5. Results for the individual groups are shown in Table 1.

**Figure 1:**
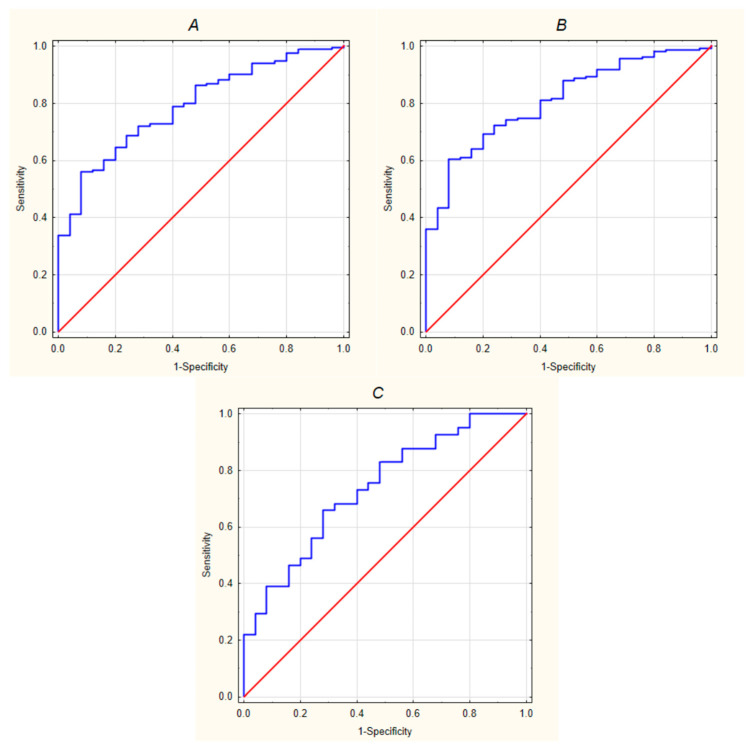
The diagnostic performance of CXCL12 concentration (pg/mL) in cerebrospinal fluid (CSF) in differentiating patients from controls and according to etiology: (**A**) whole study group, (**B**) tick-borne encephalitis (TBE), (**C**) aseptic meningitis.

**Figure A1:**
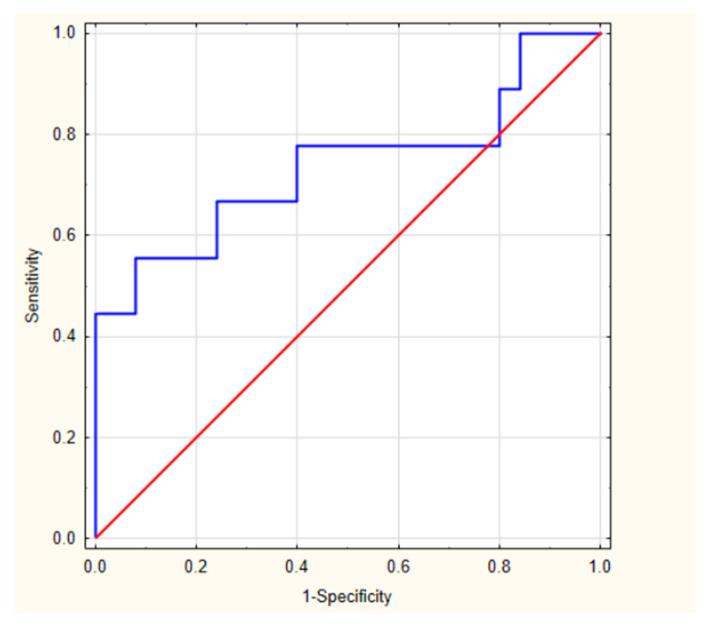
The diagnostic performance of CXCL12 concentration (pg/mL) in cerebrospinal fluid (CSF) in differentiating neuroborreliosis from the control group by ROC curves.

**Figure A2:**
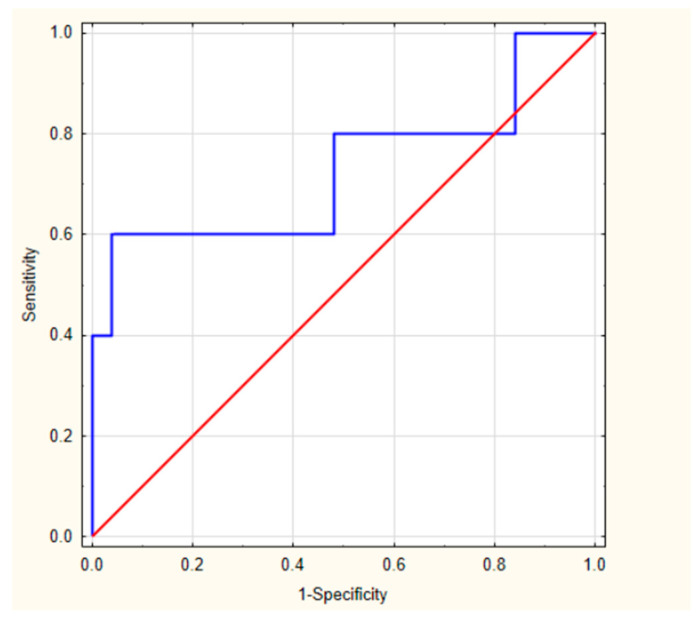
The diagnostic performance of CXCL12 concentration (pg/mL) in cerebrospinal fluid (CSF) in differentiating purulent meningitis from the control group by ROC curves.

CXCL12 concentrations were significantly higher in patients with neuroinfections compared with controls (*p* < 0.001), with median in the study group being 2201.06 pg/mL and in controls 1292.99 pg/mL. ROC curve analysis indicated that CXCL12 concentration has statistically significant and good discriminative ability between control and study group (AUC = 0.791; 95% CI 0.711–0.871; *p* <0.001) (Figure 1A). CXCL12 levels were also significantly higher when comparing individual groups with controls, except for the purulent meningitis group, where the effect was not statistically significant, likely due to the small sample size. Detailed results are presented in Table 2. Separate ROC curve analyses were performed for each group (Figure 1B,C). The results for NB and purulent meningitis are provided in Table A1 along with their ROC curves (Figure A1 and Figure A2). Owing to the small sample size, they should be interpreted with caution and regarded as preliminary findings.

**Figure 2:**
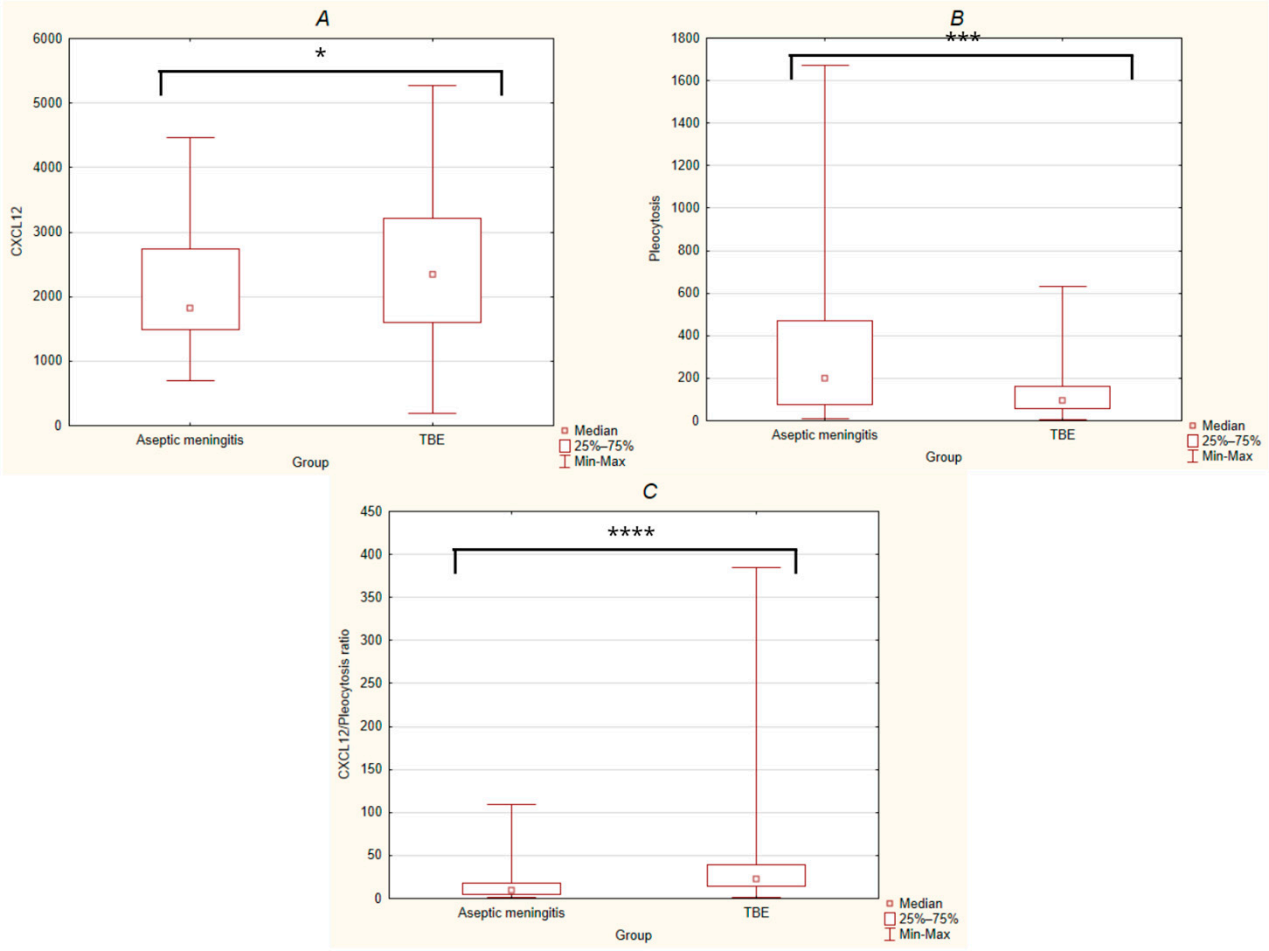
Comparison of CXCL12 concentration (**A**), pleocytosis (**B**) and CXCL12/pleocytosis ratio (**C**) between tick-borne encephalitis (TBE) and aseptic meningitis. A statistically significant difference in concentrations was observed (* = *p* < 0.05, *** = *p* < 0.001, **** = *p* < 0.0001).

**Figure 3:**
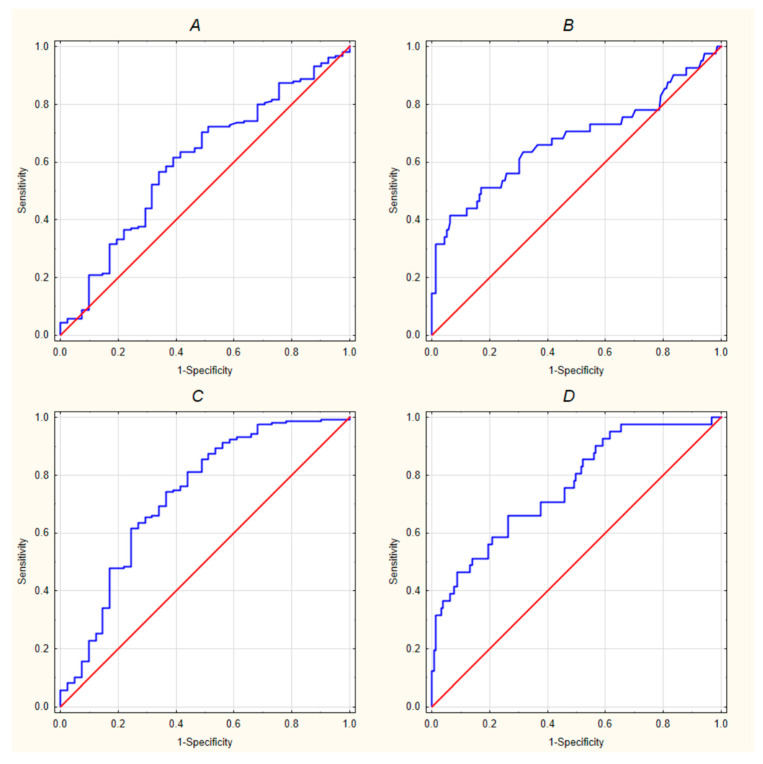
Diagnostic performance for differentiating tick-borne encephalitis from aseptic meningitis, using ROC curves: CXCL12 alone (**A**), pleocytosis alone (**B**), the CXCL12/pleocytosis ratio (**C**), and a combined ROC model including CXCL12 and pleocytosis (**D**).

Taking into account a practical clinical perspective, the initial step of the analysis focused on comparing patients with TBE to those with aseptic meningitis. Chemokine CXCL12 concentrations were significantly higher in the TBE group compared to aseptic meningitis group (Mann–Whitney U test, U = 2588, Z = 2.03, *p* < 0.05) (Figure 2A). ROC curve analysis showed that CXCL12 levels had statistically significant but weak ability to differentiate TBE from aseptic meningitis (AUC = 0.603, 95% CI: 0.506–0.70; *p* < 0.05) (Figure 3A). Pleocytosis was also statistically lower in the TBE group compared to patients with aseptic meningitis (Mann–Whitney U test, U = 2100.5, Z = 3.51, *p* < 0.001) (Figure 2B) and showed better discriminative performance (AUC = 0.678, 95% CI = 0.57–0.786) (Figure 3B). The CXCL12/pleocytosis ratio was statistically higher in the TBE group (Mann–Whitney U test, U = 1769, Z = −4.51, *p* < 0.0001) (Figure 2C) and revealed even better differentiating performance (AUC = 0.729, CI = 0.633–0.825). A combined ROC model including both CXCL12 and pleocytosis further improved diagnostic accuracy (AUC = 0.761, 95% CI = 0.678–0.845) (Figure 3C,D).

**Figure A3:**
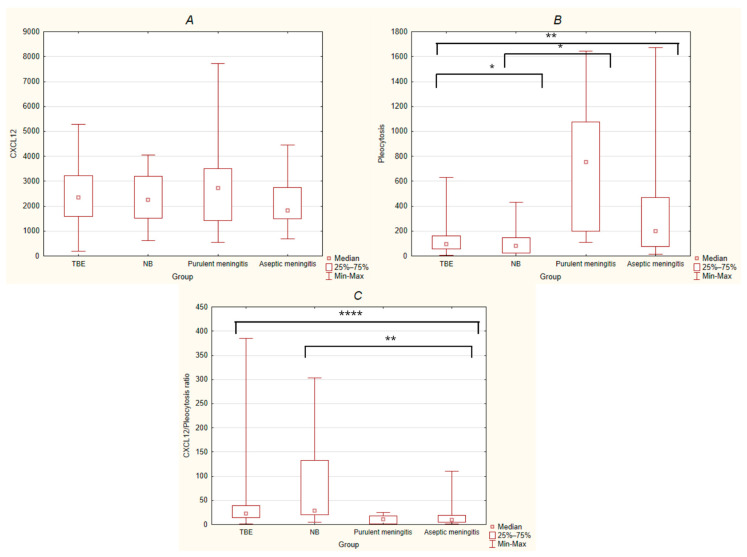
Comparison of CXCL12 concentration (**A**), pleocytosis (**B**) and CXCL12/pleocytosis ratio (**C**) between different etiologies: tick-borne encephalitis (TBE), aseptic meningitis, purulent meningitis, and neuroborreliosis (NB). A statistically significant difference in concentrations was observed (* = *p* < 0.05, ** = *p* < 0.01, **** = *p* < 0.0001).

**Figure A4:**
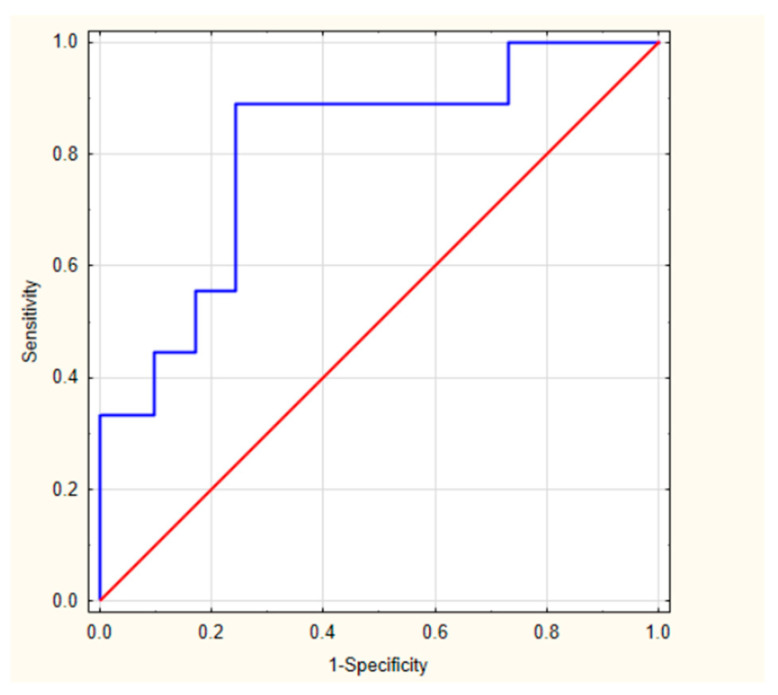
Diagnostic performance for differentiating neuroborreliosis from aseptic meningitis using ROC curves: CXCL12/pleocytosis ratio.

Subsequently, a comparative analysis across all four etiological groups was performed (Figure A3). The Kruskal–Wallis test did not reveal statistically significant differences in CXCL12 concentrations among groups. This finding should be interpreted with caution as a preliminary result with limited sensitivity, likely due to the small sample sizes of the bacterial groups (NB and purulent meningitis). However, the same test showed statistically significant differences in pleocytosis between different etiologies (*p* < 0.0001); therefore, additional post hoc comparisons with Bonferroni correction were done confirming that the TBE group differs significantly from aseptic meningitis (*p* < 0.01) and purulent meningitis group (*p* < 0.05), whereas NB group differs significantly from purulent meningitis group (*p* < 0.05). The rest of the differences were statistically insignificant. Furthermore, analysis of the CXCL12/pleocytosis ratio across four groups revealed significant differences between etiologies in the Kruskal–Wallis test (*p* < 0.0001). Post hoc pairwise comparisons with Bonferroni correction showed that the aseptic meningitis group differed significantly from both the TBE group (*p* < 0.0001) and the NB group (*p* < 0.01). Due to the statistically significant difference in the CXCL12/pleocytosis ratio between the aseptic meningitis and NB groups, an ROC curve analysis was performed, which demonstrated very good discriminative performance (AUC = 0.808, 95% CI = 0.649–0.966) (Figure A4). While the results for the TBE and aseptic meningitis groups are supported by large study cohorts, the findings for the NB and purulent meningitis groups are based on a limited number of patients and remain preliminary; therefore, these results should be interpreted with caution.

No statistically significant difference in CXCL12 levels was found between patients with viral (*N* = 200) and bacterial infections (*N* = 14) (*p* > 0.05). 

### 2.3. Changes in CXCL12 Levels over Time

In 53 patients who also underwent a second assessment of CXCL12 in CSF, the mean number of days between first and second measurement was 25.4 ±12.99 days. Chemokine concentrations differed significantly between the first and second measurements (Wilcoxon signed-rank test, Z = 1.996, *p* < 0.05). The median difference in CXCL12 levels was 623.318, with a downward trend. Results within individual groups were not statistically significant. 

### 2.4. Correlations with CSF Cellular Parameters

Spearman rank correlation analysis showed a statistically significant, positive, but weak relationship between the CXCL12 concentration and pleocytosis in the whole study group, TBE group and in aseptic meningitis group, where correlation was the highest (rho = 0.463; *p* < 0.01). Moreover, regression analysis showed that CXCL12 is a significant predictor of pleocytosis in the other viral group, explaining approximately 14% of its variability (R^2^ = 0.1395; *p* < 0.05), indicating the involvement of additional factors influencing this parameter. 

In the overall study group, the TBE group and the aseptic meningitis group, Spearman’s rank correlation analysis demonstrated a weak positive correlation between CXCL12 concentration and the absolute lymphocyte count in CSF. This association was the highest in the aseptic meningitis group (rho = 0.419; *p* < 0.01). Regarding the absolute neutrophil count in the CSF, a weak positive correlation was found with CXCL12 concentration in both the overall study group and the TBE group. Aseptic meningitis was the only group with statistically significant moderate positive correlation between CXCL12 concentration and the absolute monocyte count in CSF (rho = 0.448; *p* < 0.01). All correlations mentioned above are presented in Table 3.

### 2.5. CXCL12 Correlations with QAlb and CSF Protein

Among 165 patients with albumin quotient (Q_Alb_) measured, 136 (82.42%) Q_Alb_ exceeded the upper limit of normal. In the overall study group and in the TBE group, Spearman’s rank correlation analysis revealed a weak positive correlation between CXCL12 concentration and Q_Alb_ in CSF.

Similarly, in the overall study group and in the TBE group, Spearman’s rank correlation analysis demonstrated a weak positive correlation between CXCL12 concentration and CSF protein levels (mg/dL). All correlations mentioned above are presented in Table 3.

### 2.6. Correlations with the Clinical Presentation

**Figure 4:**
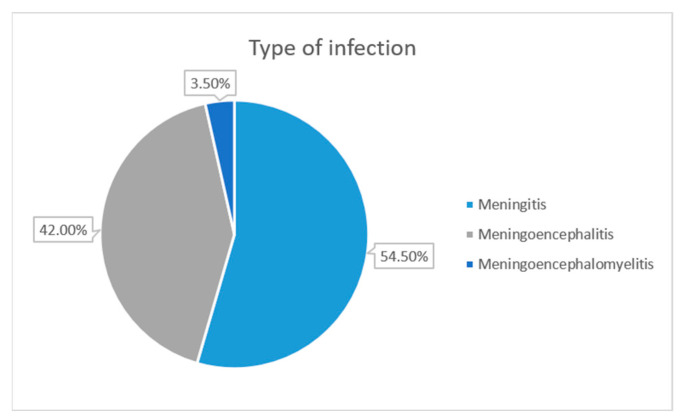
Patients from TBE and aseptic meningitis groups categorized according to the type of infection: M—meningitis, ME—meningoencephalitis, MEM—meningoencephalomyelitis.

**Figure 5:**
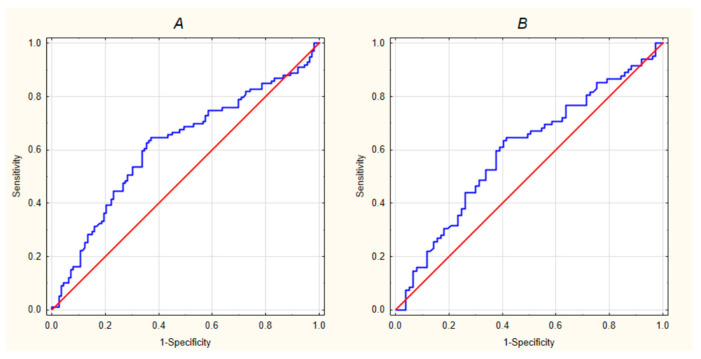
The ability of CXCL12 to discriminate between mild and severe cases in the whole study group (**A**) and in tick-borne encephalitis group (**B**) using ROC curves.

Moving to the analysis of infection types and their severity, M was diagnosed in 109 patients (54.5%) (median age 40; 41 females and 68 males), ME in 84 patients (42%) (median age 51.5; 29 females and 55 males) and MEM in 7 patients (3.5%) (median age 52; 1 female and 6 males) (Figure 4). CXCL12 concentrations were significantly higher in patients with severe disease (meningoencephalitis/meningoencephalomyelitis) compared with mild cases (meningitis), both in the overall cohort (*p* < 0.01) and in the TBE group (*p* < 0.05). In the aseptic meningitis group no statistically significant difference in CXCL12 concentration was observed between the mild and severe forms of the disease. ROC analysis showed the limited ability of CXCL12 to distinguish between severe and mild disease, both in all patients (AUC = 0.615, 95% CI = 0.538–0.692, *p* < 0.01) and in the TBE subgroup (AUC = 0.596 95% CI = 0.508–0.685, *p* < 0.05) (Figure 5).

**Figure 6:**
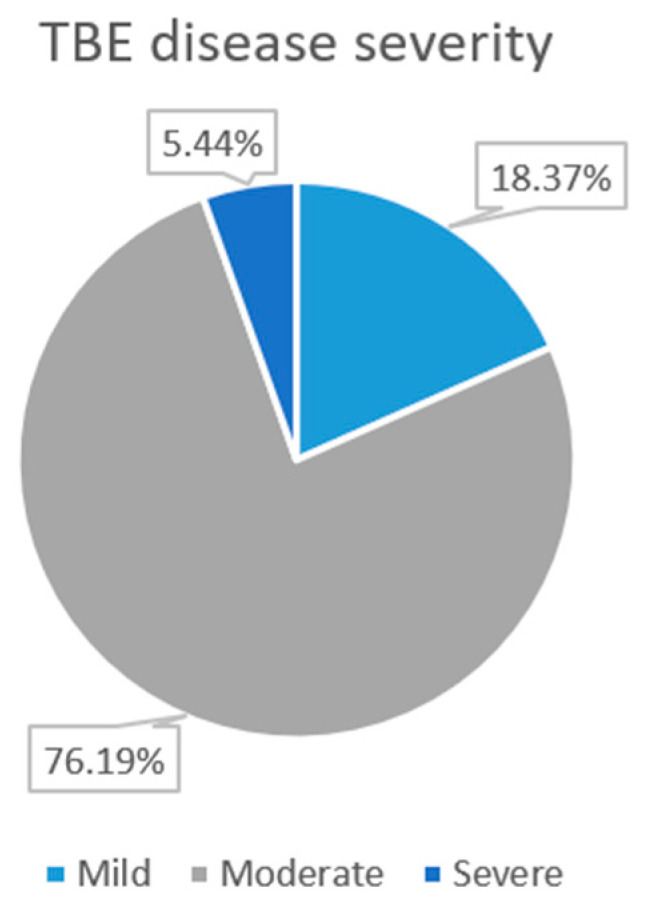
Patients included in tick-borne encephalitis (TBE) group classified by disease severity score using Bogovič scale.

In 147 patients with TBE, the disease severity was assessed quantitatively using a scale proposed by P. Bogovič [24]. In the 0–42 points range, the mean score was 13.06. The patients who received 0–8 points were qualified as having mild disease, those with 9–22 points as moderate and those with >22 points as severe. A total of 27 patients (18.37%) were qualified into mild, 112 into moderate (76.19%) and 8 (5.44%) into severe disease (Figure 6). The Spearman correlation analysis showed that there was no statistically significant rank correlation between CXCL12 levels and the score in the Bogovič scale (*p* > 0.05).

To further determine if there is an association between CXCL12 levels and clinical presentation, CXCL12 concentrations in the whole study group were compared across subgroups defined by the presence or absence of specific symptoms (disturbances of consciousness, paresis, cerebellar signs and tremor). No significant differences were observed, except for patients presenting with tremor in whom significantly higher CXCL12 levels were observed. (Mann–Whitney U test: U = 881.0; Z = −2.45; *p* = 0.014).

An analysis was performed to determine whether there was a statistically significant correlation between chemokine concentration and disease outcome, which was categorized as lack of symptoms, subjective complaints, and neurological abnormalities. No statistically significant correlation was found.

## 3. Discussion

The analysis of CXCL12 concentrations in relation to CSF cellular parameters demonstrates differences depending on the etiology of neuroinfection. Although CXCL12 concentrations tended to be higher in purulent meningitis and TBE (Table 1), their relationships with cellular parameters differed substantially (Table 3).

In TBE, relatively elevated CXCL12 levels coincided with moderate pleocytosis, showing a clear predominance of lymphocytes. The association was stronger with overall pleocytosis and lymphocyte counts than with neutrophils, which is consistent with the specific activity of CXCL12 mainly on mononuclear cells. This may indicate a role for CXCL12 in regulating controlled lymphocyte migration, which is consistent with results published in the literature [25,26,27].

Purulent meningitis etiology characterized by both the highest CXCL12 concentrations and the highest pleocytosis, with a strong predominance of neutrophils. However, the lack of significant correlations between CXCL12 and cellular components in this group may suggest that elevated CXCL12 represents a marker of global inflammatory activation and BBB disruption rather than being specifically linked to neutrophil recruitment. However, a relatively small number of participants might have affected the ability to demonstrate statistical significance.

The group of aseptic meningitis exhibited the lowest CXCL12 concentrations, along with relatively high, predominantly lymphocytic pleocytosis. Importantly, this group was characterized by the strongest correlations between CXCL12 and pleocytosis among all etiologies, as well as between CXCL12 and lymphocytes and between CXCL12 and monocytes, suggesting an even closer association with mononuclear-driven inflammation than in TBE. This may suggest that, in aseptic meningitis, CXCL12 expression is more directly linked to mononuclear cells recruitment, although other chemokine pathways likely remain important. The correlation between the chemokine and neutrophils in this group was not statistically significant, and in the TBE group it was lower than the correlation with lymphocytes. These findings are consistent with previous studies, demonstrating that CXCL12 plays a key role in regulating the migration of mononuclear cells rather than acting as a general chemoattractant for all leukocyte populations [3,4,28].

NB presented with relatively elevated CXCL12 levels, low pleocytosis and absence of neutrophils, accompanied by a mixed lymphocyte–monocyte response. No significant correlations between CXCL12 and CSF parameters were observed. However, a limitation was the small size of the groups with bacterial infections, which made it difficult to confirm statistical significance. These results are consistent with findings by Kowarik et al. and Moniuszko et al. [18,29]. Moreover, Moniuszko et al. demonstrated that other chemokines such as CXCL13, CXCL10, and CXCL11 play a more relevant role in the inflammatory response in NB than CXCL12 [18]. Altogether, the presented results suggest that CXCL12 concentration is not a simple reflection of the intensity of pleocytosis or cellular composition but may be associated with qualitative differences in the immune response and leukocyte trafficking.

Analysis of discriminatory performance of CXCL12 concentration in differentiating individual etiological groups from the control group demonstrated that CXCL12 concentrations in CSF may be moderate biomarkers of the CNS inflammation in all of the investigated etiologies except from purulent meningitis, where the result was not statistically significant. However, the negative result in purulent meningitis may be due to the small sample size, so it should not be considered a definitive result. Elevated CXCL12 levels were observed across all remaining etiological groups; nonetheless, it may suggest that its expression reflects a general inflammatory response rather than a disease-specific mechanism. The literature has also reported significantly higher CXCL12 levels in neuroinfections compared to controls. This finding was observed in both NB [18,30] and TBE [19,25,27]. There is still limited information regarding bacterial as well as other viral etiologies.

As a standalone diagnostic marker, CXCL12 has a limited ability to distinguish between different etiologies. This was also demonstrated in the study by Pietikäinen et al. [30], which compared NB with TBE. In our study the CXCL12/pleocytosis ratio demonstrated better diagnostic utility than the chemokine alone, particularly in differentiating between TBE and aseptic meningitis; however, its clinical value requires further evaluation in larger NB cohorts. A combined model including both CXCL12 and pleocytosis was the most useful parameter in differentiating TBE and aseptic meningitis.

Difference in CXCL12 concentration over time between two measurements, resulting from the resolution of the inflammatory process associated with the applied treatment, suggests that CXCL12 is linked to the active phase of the inflammatory process, and its level decreases as the inflammation resolves. Even though this effect was not statistically significant within individual etiological groups, likely due to limited sample sizes, it remains clinically relevant. Interestingly, previous studies by Zajkowska et al. demonstrated that chemokine levels may persist despite clinical improvement, indicating ongoing subclinical inflammation within the CNS. Taken together, these findings suggest that CXCL12 reflects dynamic but not necessarily fully resolved inflammatory processes [19].

As our study showed, among patients with TBE for whom the Bogovič scale was developed, the majority were classified as having a moderate disease course. CXCL12 concentration was not associated with the severity score, which may indicate that the level of this cytokine is not a reliable predictor of disease severity. In contrast, when disease form was assessed according to the classification into M, ME or MEM groups, CXCL12 levels were statistically significantly higher in severe clinical presentation (ME and MEM), both in the combined TBE and aseptic meningitis groups and in the TBE group alone. This suggests that CXCL12 concentration is not a good indicator of disease severity as assessed with the Bogovic scale, but it may help differentiate between M, ME and MEM forms of the disease. Nevertheless, its diagnostic contribution appears to be limited, and additional parameters would be required. According to Czupryna et al., CSF YKL-40 may serve as a potential biomarker for distinguishing between tick-borne M and ME, as its concentration was significantly higher in patients with M [31]. Recent data suggest that CSF biomarkers may improve risk stratification, as 32 metabolomic markers including S-adenosylmethionine, fructose-1,6-bisphosphate and phosphoenolpyruvate were shown to differentiate encephalitis from meningitis in TBE patients [32]. Assessing TBE severity is complex as it is determined by multiple clinical and laboratory factors. Factors associated with a more severe disease include prior vaccination, older age, immunosupression, higher pleocytosis and blood leukocyte count, and higher serum C-reactive protein levels [16,33].

Because CXCL12, dependent on its local concentration and coexisting factors [5], may have both protective and disruptive effects on BBB, we have analyzed its association with the biochemical markers of BBB disruption. The value of QAlb and CSF protein concentration showed a weak positive correlation with CXCL12 concentration in the overall study group as well as in TBE. This may suggest a limited association between CXCL12 and BBB permeability or inflammatory processes. Due to the weak correlations and inconsistency across etiological groups, it cannot be considered a reliable marker of blood–brain barrier disruption.

Among the limitations of our study, we can indicate imbalance in the sizes of the analyzed subgroups, particularly the small number of patients with NB and purulent meningitis etiologies. The limited number of these groups led to increased standard deviations in ROC curve analyses and broader confidence intervals. Therefore, further studies in larger cohorts are necessary to evaluate the true potential of CXCL12 as a diagnostic tool in differentiating between these etiologies. Another limitation is that only a subset of patients had a second measurement of CXCL12 concentration in the CSF performed. Furthermore, the time intervals between repeated measurements differed between patients. We would like to emphasize the need for further validation of CXCL12 as a biomarker across different etiologies of neuroinfections in future studies.

## 4. Materials and Methods

### 4.1. Study Group

A group of 214 patients hospitalized in the Department of Infectious Diseases and Neuroinfectious of the Medical University of Bialystok, Poland, between 2019 and 2024 were included in the study and divided according to etiology.

Inclusion criteria for patients included in the TBE group were: 1. Presence in a tick-endemic area within the last 3 weeks or a recent tick bite; 2. Symptoms suggestive of meningitis and/or encephalitis; 3. Cerebrospinal fluid (CSF) pleocytosis ≥ 5/µL; 4. Presence of anti-TBEV IgM antibodies in CSF. Inclusion criteria for patients with NB included criteria 2 and 3 along with possible or confirmed tick bites within previous 6 months and intrathecal production of antibodies against Borrelia burgdorferi sensu lato. TBE and NB sets of inclusion criteria were established in accordance with the European case definition criteria [34]. Inclusion in the purulent meningitis group was made in cases where the CSF showed a purulent character and was confirmed by CSF culture or based on the purulent nature of the CSF findings together with the clinical presentation. Patients in whom antibodies against TBEV or Borrelia burgdorferi were not detected nor had purulent CSF with positive cultures, and who met criteria 2 and 3, were diagnosed with aseptic meningitis. The clinical presentation in this group of patients was most consistent with a viral infection. Contact with a tick was not required to qualify for this group. Additionally, patients diagnosed with herpes simplex encephalitis, confirmed by polymerase chain reaction (PCR) testing of CSF, were included in the aseptic meningitis group.

The control group included patients hospitalized with a suspicion of meningitis in whom infection of CNS had been excluded based on noninflammatory CSF samples obtained through lumbar puncture. The CSF cell count and protein levels were within the normal range and differed significantly from those observed in the study group.

Additionally, patients from TBE and aseptic meningitis groups were divided based on neurological symptoms and altered consciousness into mild (meningitis) and severe groups (meningoencephalitis/meningoencephalomyelitis). Meningitis (M) was diagnosed based on inflammatory parameters in CSF and lack of neurological symptoms. When inflammation in CSF was accompanied by focal neurological symptoms and/or altered consciousness, meningoencephalitis (ME) was diagnosed. The diagnosis of meningoencephalomyelitis (MEM) was made when the presence of flaccid limb paralyses was observed.

In 147 patients with TBE, the disease severity was assessed quantitatively using a scale proposed by P. Bogovič et al. [24].

All the patients gave informed written consent for participation in the study.

### 4.2. Material Acquisition and Handling

The paired CSF and blood samples were obtained on admission day. Subsequently, the samples were kept at 5 °C for a maximum of 24 h prior to processing. For cytokine analysis, 1.0 mL of CSF was drawn into a sterile plastic tube and frozen on the day of collection, stored at −40 °C, and thawed immediately before analysis at the end of the study period.

### 4.3. Basic Laboratory Examinations

Anti-TBEV IgM and IgG antibodies were assessed in both serum and CSF samples collected at admission as well as on follow-up in originally seronegative patients, using EuroImmun Anti-TBE virus ELISA kits (Lübeck, Germany) following the standard procedure in a hospital diagnostic laboratory. According to the manufacturer, the serum anti-TBEV IgM assay demonstrates 100% sensitivity and specificity, based on evaluation in a cohort of 129 patients. Negative results obtained at admission were rechecked prior to discharge or during follow-up visits, and only patients that remained negative were definitively assigned to aseptic meningitis group.

Screening serological testing for Lyme disease was performed using the ELISA test by Biomedica, and intrathecal synthesis was verified using the Borrelia EcoLine immunoblot test.

The CSF pleocytosis with differential and protein levels as well as albumin concentrations in serum and CSF were measured with standard laboratory techniques. Albumin quotient (QAlb) was calculated in 165 patients.

### 4.4. Cytokine Concentration

The concentration of CXCL12 was measured from a separate CSF sample using a commercial ELISA kit from R&D Systems (Minneapolis, MN, USA), following the manufacturer’s instructions. These measurements were performed exclusively in CSF, as the assay was not validated for serum samples. The concentration of cytokine was expressed in pg/mL. In 53 patients who underwent a second lumbar puncture due to diagnostic reasons, the second measurement of CXCL12 concentration in CSF was done. The mean number of days between first and second measurement was 25.4 ± 12.99 days. Among them, 42 represented TBE group, 8 aseptic meningitis group and 3 NB group.

### 4.5. Statistical Analysis

Subsequently, the statistical analysis was made using the Statistica 14 software. The normality of distribution was assessed by the Shapiro–Wilk test. Mann–Whitney U test for two-group comparison, non-parametric Kruskal–Wallis ANOVA for multiple group comparisons with additional post hoc comparisons with Bonferroni correction, Wilcoxon pair test for two-variable comparisons, and Spearman’s rank-order correlation for correlation analysis were used. The analysis of receiver operating characteristic (ROC curve) was made for selected parameters. *p* < 0.05 was considered statistically significant. Linear regression analyses were performed to assess associations between variables.

## 5. Conclusions

The results suggest that CXCL12 may be one of the chemotactic factors responsible for the influx of lymphocytes into CNS in TBE. In aseptic meningitis it may be even more closely associated with mononuclear cell driven inflammation than in TBE.

Based on ROC curve analysis, we conclude that CXCL12 concentrations in CSF may serve as a useful biomarker of general neuroinflammation rather than a marker of specific neuroinfectious etiologies. CXCL12 demonstrated moderate to good discrimination between patients with neuroinfections and controls, whereas its discriminative performance was generally lower across individual etiological groups.

When it comes to distinguishing different etiologies of neuroinfections, CXCL12 alone has limited ability. The improved diagnostic accuracy observed in the combined model with pleocytosis indicates that CXCL12 may provide additional value as part of a multimarker approach rather than as a standalone biomarker.

Based on the presented results and given the complexity of neuroinflammatory processes, we emphasize the need for developing multifactorial prognostic models, in which CXCL12 could be considered as one of the variables.

## Figures and Tables

**Table 1:** Cerebrospinal fluid parameters in the whole study group and different etiologies. TBE—tick-borne encephalitis; NB—neuroborreliosis.

Etiology	Median CXCL12 Concentration (pg/mL)	Median Pleocytosis (Cells/µL)				Median Q_Alb_	Median CSF Protein Levels (mg/dL)
		Total	Lymphocytes	Neutrophils	Monocytes		
Whole study group	2201.06	102.5	62.37	12.96	16.85	10.73	69
TBE	2338.77	94	57.9	14.07	15.04	10.93	67.5
Aseptic meningitis	1814.52	199	118.26	7.86	31.84	9.54	69
NB	2254.57	80	49.6	0	11.92	12.01	60
Purulent meningitis	2722.71	754	66.96	203.58	33.83	-	578
Control group	1292.99	2	-	-	-	-	29

**Table 2:** Discriminatory performance of CXCL12 concentration in differentiating individual etiological groups from the control group assessed by ROC curve analysis. TBE—tick-borne encephalitis.

Etiology	AUC	95% CI	*p*-Value	Interpretation
Whole study group	0.791	0.711–0.871	<0.001	Good discrimination
TBE	0.81	0.731–0.888	<0.001	Good discrimination
Aseptic meningitis	0.737	0.615–0.858	<0.001	Moderate discrimination

**Table 3:** Correlation between CXCL12 concentration and various factors, including pleocytosis, lymphocytes, neutrophils, and monocytes in CSF, as well as QAlb and CSF protein levels, across different etiologies. The presented values are Spearman’s rho coefficients shown only for statistically significant results (* = *p* < 0.05, ** = *p* < 0.01, *** = *p* < 0.001), NS—non-significant.

	Whole Study Group	TBE	Aseptic Meningitis
Pleocytosis	0.228 ***	0.227 **	0.463 **
Lymphocytes in CSF	0.241 ***	0.242 **	0.419 **
Neutrophils in CSF	0.183 **	0.178 *	NS
Monocytes in CSF	NS	NS	0.448 **
Q_Alb_	0.195 *	0.202 *	NS
CSF protein levels	0.231 ***	0.234 **	NS

## Data Availability

The datasets used and/or analyzed during the current study are available from the corresponding author on reasonable request.

## Abbreviations

The following abbreviations are used in this manuscript:

TBE	Tick-borne encephalitis
CSF	Cerebrospinal fluid
BBB	Blood-brain barrier
CNS	Central nervous system
NB	Neuroborreliosis
PCR	Polymerase chain reaction
M	Meningitis
ME	Meningoencephalitis
MEM	Meningoencephalomyelitis
QAlb	Albumin quotient
ROC	Receiver operating characteristic
ELISA	Enzyme-Linked Immunosorbent Assay

## Appendix A

Preliminary results of using CXCL12 as a diagnostic marker to distinguish NB and purulent meningitis from controls.

**Table A1:** Discriminatory performance of CXCL12 concentration in differentiating neuroborreliosis (NB) and purulent meningitis groups from the control group assessed by ROC curve analysis.

Etiology	AUC	95% CI	*p*-Value	Interpretation
NB	0.738	0.514–0.962	<0.05	Moderate discrimination
Purulent meningitis	0.728	0.423–1	>0.05	NS

## Appendix B

Comparison of CXCL, pleocytosis, and their ratio between all studied groups.

## Appendix C

Preliminary results of using the CXCL12/pleocytosis ratio to differentiate neuroborreliosis from aseptic meningitis.
